# Sestrin2 is Induced Upon Cellular Stress but Has No Effect on Myotube Size or Amino Acid Sensing in C2C12 Myotubes

**DOI:** 10.1111/boc.70040

**Published:** 2025-11-06

**Authors:** Jiani Qian, Stephanie D. Gagnon, Vladimir Belhac, Carl J. Hulston, Neil R. W. Martin

**Affiliations:** ^1^ School of Sport, Exercise and Health Sciences Loughborough University Leicestershire UK; ^2^ Randall Centre for Cell and Molecular Biophysics King's College London Guy's Campus London SE1 1UL UK; ^3^ Perelman Centre for Cellular and Molecular Therapeutics Children's Hospital of Philadelphia Pennsylvania USA; ^4^ School of Sport, Exercise and Rehabilitation Sciences University of Birmingham Birmingham UK

**Keywords:** ER stress, mTORC1, myotubes, nutrient sensing, skeletal muscle

## Abstract

Sestrins are a stress‐inducible family of proteins that function in cell survival and nutrient sensing through their regulation of mTORC1. Muscle wasting is associated with cellular stress, but to date, there is limited in vitro research investigating sestrins in skeletal muscle cells. Here we use C2C12 myotubes to understand how sestrin proteins (sestrin 1–3) are regulated by different forms of cellular stress linked to muscle wasting conditions. Furthermore, since sestrin2 is a well‐characterised protein but is lowly expressed in muscle tissue in the absence of stress, we also aimed to determine if silencing this protein impacted parameters of muscle growth or nutrient sensing by mTORC1 under basal conditions. Incubating C2C12 myotubes with the endoplasmic reticulum (ER) stress‐inducing agent tunicamycin, or a high concentration (1000 µM) of hydrogen peroxide (H_2_O_2_), increased sestrin2 protein levels with no change in sestrins 1 or 3. This increase was temporally associated with increased ER stress markers *Ddit3* mRNA and ATF4 protein levels, and could be blocked by approximately half when myotubes were co‐incubated with H_2_O_2_ and the ER‐stress inhibitor 4‐Phenylbutyrate. siRNA silencing of sestrin2 blunted the phosphorylation of the mTORC1 effector S6K1, but did not acutely influence protein synthesis or myotube size. Similarly, silencing sestrin2 did not affect mTORC1 signalling in response to nutrient deprivation. These data indicate that sestrin2 is stress‐inducible and may play a role in protecting skeletal muscle from ER stress, but is less important in regulating mTORC1 and nutrient sensing in unstressed/basal conditions.

## Graphical abstract

Endoplasmic reticulum stress is associated with muscle wasting, and specifically induces sestrin2 levels within C2C12 myotubes, but not sestrins 1 or 3. Silencing sestrin2 in myotube cultures does not acutely alter myotube size, or the ability of mTORC1 to sense the presence of amino acids. Sestrin2 is likely an important regulator of cellular health under stresses conditions but does not influence anabolism in unstressed muscle.

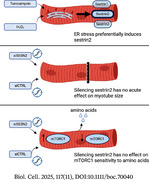

## 1 Introduction

Maintenance of skeletal muscle mass throughout the life course is crucial for health. Skeletal muscle wasting such as occurs during ageing, immobilisation and disease leads to a reduced quality of life (Hanna et al. 2022; Trombetti et al. 2016), metabolic dysfunction (Srikanthan and Karlamangla 2011) and increased mortality (Ruiz et al. 2008), and as such is considered a major public health concern. Cellular stresses such as inflammation, oxidative and endoplasmic reticulum (ER) stress are prevalent in conditions characterised by muscle loss, and when these stresses are exacerbated, they result in muscle wasting in pre‐clinical models (Baker et al. 2018; Qiu et al. 2018; Wang et al. 2023). For suitable therapeutics for muscle wasting to be generated in the future, it is imperative that the scientific understanding of the mechanisms linking stress signals to muscle wasting is better understood.

Sestrins are an evolutionary conserved family of proteins consisting of three mammalian homologs (sestrins 1–3) which appear to serve several cellular functions, including nutrient sensing (Wolfson et al. 2016) and cell survival (Ben‐Sahra et al. 2013; Ding et al. 2016; Saveljeva et al. 2016; Zhao et al. 2014). Sestrins are robustly and distinctly induced by cellular stress across a range of cell types. For example, mouse macrophages upregulate sestrin2, but not sestrin1 mRNA in response to lipopolysaccharide‐induced inflammation (Kim et al. 2015), whereas in RKO cancer cells, sestrins 1 and 2 are increased following hydrogen peroxide treatment (Budanov et al. 2004). In addition, endoplasmic reticulum (ER) stress and the ensuing unfolded protein response (UPR) apparently increase sestrin2 expression specifically in several cell types (Ding et al. 2016; Park et al. 2014) through its transcription factor ATF4 (Brüning et al. 2013; Ding et al. 2016). In skeletal muscle, sestrin proteins are increased by exercise (Crisol et al. 2018; Lenhare et al. 2017; Liu et al. 2015) and downregulated by inactivity (Segalés et al. 2020). Moreover, muscle loss caused by denervation is associated with specific induction of sestrin2, with no effect on sestrins 1 or 3 (Yang, Xue, et al. 2021), which may also be related to ER stress (Brown et al. 2018; Yang, Xue, et al. 2021). However, the effect of specific stresses that can induce muscle wasting, such as inflammation, oxidative and ER stress, on protein levels of sestrin family members has not been directly tested.

One of the most established features of sestrin proteins is their ability to regulate the mechanistic target of rapamycin complex 1 (mTORC1) signalling pathway, and indeed, this is thought to be the principal mechanism by which sestrins exert their cellular functions (Chen et al. 2022). mTORC1 is a central regulator of cellular growth, and in the presence of nutrients (primarily amino acids), it stimulates protein synthesis via the phosphorylation of its downstream substrates S6K1 and 4E‐BP1 (Ben‐Sahra and Manning 2017). Sestrins bind to and inhibit GATOR2, a protein complex that lies upstream of mTORC1 and acts as a positive regulator of its activity (Chantranupong et al. 2014; Parmigiani et al. 2014; Peng et al. 2014), and thus sestrins are thought to suppress mTORC1 signalling. The amino acid leucine, which possesses potent anabolic properties, is bound by sestrin1 and/or 2 inside the cell, which alleviates sestrin inhibition of GATOR2 and promotes mTORC1 activation (Wolfson et al. 2016). Sestrin 1 is the most abundant sestrin protein in skeletal muscle tissue (Peeters et al. 2003; Xu et al. 2019), and surprisingly, overexpression of sestrin1 in mice protects against muscle wasting observed after immobilisation through reducing hyperactive mTORC1 signalling (Segalés et al. 2020). However, to date, it is unknown how alterations in the sestrin2 proteins influence mTORC1, protein synthesis and muscle size in unstressed/basal conditions. Furthermore, leucine alleviates sestrin 1 (but not sestrin2) binding to GATOR2 to promote mTORC1 stimulation in skeletal muscle in vivo (Xu et al. 2019), suggesting that sestrin1 is more important for amino acid sensing in skeletal muscle. However, whilst knockout of all sestrin isoforms results in constitutively active mTORC1 signalling in the absence of amino acids in skeletal muscle of mice (Peng et al. 2014), overexpression of sestrin1 alone failed to dampen mTORC1 activation in rodent muscle following leucine ingestion (Xu et al. 2019). It is less clear how sestrin2 levels influence the mTORC1 response to amino acid availability in skeletal muscle cells.

The purpose of the current experiments was to explore how sestrin proteins respond to cellular stresses associated with muscle wasting in cultured skeletal muscle cells and subsequently determine the acute effect of sestrin2 silencing on mTORC1 signalling, protein synthesis and myotube size. Finally, we explored whether silencing of sestrin2 influences mTORC1 response to nutrient deprivation. Our data suggests that sestrin2 protein levels are specifically related to enhanced ER stress in skeletal muscle cells. However, even though acute silencing of sestrin2 caused reduced S6K1 phosphorylation, there was no impact on protein synthesis, myotube size or mTORC1 response to amino acid withdrawal, suggesting that sestrin2 may play an important role in protecting skeletal muscle in response to stress but plays less of a role in mTORC1 regulation and nutrient sensing in muscle cells.

## 2 Methods

### 2.1 Cell Culture

C2C12 mouse myoblasts (European Collection of Authenticated Cell Cultures, Salisbury, UK) were cultured in high glucose Dulbecco's modified Eagle's medium (DMEM; Sigma–Aldrich, Dorset, UK), supplemented with 20% foetal bovine serum (FBS Good; Pan BioTech, Aidenbach, Germany) and 1% penicillin streptomycin (P/S; Fisher Scientific, Leicestershire, UK), maintained at 37°C and 5% CO_2_. At 80% confluency, the cells were routinely passaged. The C2C12 cells used in the study were below passage 10. To induce myotube formation for experimentation,100,000 cells were seeded into 6‐well plates (Fisher Scientific), and upon reaching 95% confluency, the medium was replaced with differentiation medium (DM), composed of DMEM plus 2% horse serum (Sigma–Aldrich) and 1% P/S, which was renewed after 72 h.

To induce cellular stress, after 96 h of differentiation, myotubes were incubated for 24 h with either hydrogen peroxide (H_2_O_2_) at 250, 500 and 1000 µM to induce oxidative stress, lipopolysaccharide (LPS) at 10, 100 and 1000 ng/mL to induce inflammation, or tunicamycin (TN) at 125, 250 and 500 ng/mL to induce ER stress. Control myotubes received fresh DM at the same time as the above treatments. Chemical concentrations were based on previous literature (Baker et al. 2018; Morishima et al. 2004; Siu et al. 2009) as well as preliminary experiments, where our goal was to visually discern some signs of cell death at the highest doses used (Figure S1). In a subsequent set of experiments, myotubes were treated with 1000 µM H_2_O_2_ and co‐incubated with 5 mM of sodium phenylbutyrate (4PBA; Sigma–Aldrich) for 3, 6 and 24 h.

To investigate protein synthesis in siRNA‐treated myotubes, myotubes were incubated for 30 min with 1 µM of the aminonucleoside antibiotic puromycin prior to cell lysis and immunoblotting.

For nutrient manipulation experiments, myotubes were incubated in amino acid and serum‐free DMEM, composed of 4.5 g/L glucose (Sigma–Aldrich), 3.7 g/L sodium bicarbonate (Sigma–Aldrich) and Dulbecco's MEM without amino acids (United States Biological, Salem, Massachusetts) for 6 h.

### 2.2 siRNA Transfection

During the first 48 h of myoblasts differentiation, 1 µg/mL of cytosine β‐D‐arabinofuranoside (Ara‐C; Sigma–Aldrich) was added to DM to prevent myoblasts proliferation. The application of Ara‐C in the DM was to ensure the uptake of siRNA by the differentiated myotubes rather than the proliferating myoblasts, as well as preventing the dilution of the siRNA in proliferating cells. DM was then replaced with fresh DM without Ara‐C for a further 2 days (Mastroyiannopoulos et al. 2012). Ara‐C impaired myoblast proliferation without negatively effecting differentiation (Figure S2). Before transfection, siRNA (Ambion, Austin, Texas) against sestrin2 and scrambled sequence (NC; Ambion) were complexed with lipofectamine RNAiMAX (Invitrogen, Leicestershire, UK) in Opti‐MEM (Gibco, Leicestershire, UK). The sequence of siRNA against sestrin 2 used in this thesis is F = GCUGUGGAAUACUUCCUGAtt and R = UCAGGAAGUAUUCCACAGCca. A total of 250 µL of complexes, were then added into 2.5 mL of fresh DM to give a concentration of 10 nM of NC or siRNA. Complexes were incubated for 24 h after which the media was removed, and cells treated accordingly.

### 2.3 Immunoblotting

Cell lysates were homogenised in 200 µL of RIPA buffer (Fisher Scientific) with Halt protease and phosphatase inhibitor cocktail (Fisher Scientific) and centrifuged at 13,000 × *g* for 10 min to remove insoluble material. Protein concentrations in the supernatants were determined using Pierce 660 nm Protein Assay Reagent (Fisher Scientific), and were subsequently mixed with 4× laemmli sample buffer (Bio‐Rad, Hemel Hempstead, UK) and 2‐mercaptoethanol (Sigma–Aldrich), and boiled for 5 min at 95°C. Equal volumes of protein (10 µg) were loaded into 4%–15% Mini‐PROTEAN precast gels (Bio‐Rad) and separated by electrophoresis at 150 V for 45 min. The separated proteins were transferred onto 0.2 µm polyvinylidene difluoride (PVDF) membranes (Bio‐Rad) at a constant current of 0.3 A for 90 min. PVDF membranes were washed three times in TBS containing 0.1% Tween20 (TBST) and blocked in either 5% non‐fat milk (Bio‐Rad) or 5% bovine serum albumin (BSA; Fisher Scientific) diluted in TBST for 60 min at 4°C. After three further TBST washes the membranes were incubated overnight at 4°C with primary antibody as follows: sestrin1 (1:2000 in 5% milk; Abcam, Cambridge, UK, ab134091), sestrin2 (1:2000 in 5% milk; Abcam, ab178518), sestrin3 (1:2000 in 2% BSA; Abcam, ab97792), phospho‐S6K1^Thr389^ (1:1000 in 2% milk; Cell Signaling Technology, Massachusetts, USA, #9234), S6K (1:1000 in 2% milk, Cell Signaling Technology, #2708), phospho‐rpS6^Ser235/236^ (1:5000 in 2% milk; Cell Signaling Technology, #4858), rpS6 (1:5000 in 2% milk, Cell Signaling Technology, #2217) phospho‐4E‐BP1^Thr37/46^ (1:2000 in 2% milk; Cell Signaling Technology, #2855), 4E‐BP1 (1:5000 in 2% milk, Cell Signaling Technology, #2855), CREB‐2/ATF4 (1:500 in 2% milk; Santa Cruz Biotechnology, Dallas, Texas, sc‐390063) and anti‐puromycin (1:5000 in 1% BSA; Merck, Dorset, UK, #2830984). Membranes were then washed three times with TBST and incubated for 1 h at room temperature in anti‐rabbit IgG HRP‐conjugated secondary antibody (Cell Signalling Technology) at a concentration of 1:2000 in 5% milk, 2% milk or 2% BSA, consistent with the primary antibodies dilution. Anti‐mouse IgG HRP‐conjugated secondary antibody (Cell Signalling Technology) was used for ATF4 and puromycin detection at a concentration of 1:2000 in 2% milk. After three final TBST washes, the membranes were incubated in the dark for 5 min with enhanced chemiluminescence reagents (Clarity Western ECL Substrate, Bio‐Rad). The signals were captured and quantified within the linear range of detection on the Chemidoc XRS system (Bio‐Rad) using Quantity One image software (Version 4.6.8, Bio‐Rad). Protein phosphorylation was normalised to protein loading through Coomassie Blue staining and subsequent selection of a protein band (Bass et al. 2017) and expressed as a fold change relative to the control condition.

### 2.4 RNA Extraction and RT‐qPCR

Total RNA was isolated using TRIzol (Invitrogen) according to the manufacturer's instructions and re‐suspended in 50 µL of RNA storage solution (Invitrogen). RNA concentration and quality were measured by UV spectroscopy at optical densities of 260 and 280 nm with nanodrop 2000 (Fisher Scientific). RT‐qPCR was conducted in triplicate in 384 well plates using 10 µL reaction volumes containing 5 µL of Precision PLUS OneStep qRT‐PCR Master Mix (Primerdesign, Chandlers Ford, UK), 0.5 µL of primer at a final concentration of 300 nM and 25 ng of RNA diluted in 4.5 µL of RNase free water. The primers were designed, validated and provided by Primerdesign and the sequences are shown in Table 1. RT‐qPCR was performed on a Viia7 thermal cycler (Applied Biosystems/Fisher Scientific), which was programmed to perform the following: 10 min at 55°C (reverse transcription), 2 min at 95°C (enzyme activation), followed by 40 cycles of 95°C for 10 s and 60°C for 60 s. Fluorescence was detected at the end of each cycle and data were analysed using the 2^(−ΔΔC^_T_^)^ method using *Polr2b* as a reference gene and each treatment control construct from each experiment as a calibrator.

**TABLE 1:** Primer sequences used in this study.

Target mRNA	Primer sequence 5′–3′	Product length	NCBI reference sequence
*Sesn1*	Fwd: TTGGCTGAAGTGCTGCTACC Rvs: ACACGGTCTTGACTGAGCTG	129	NM_00116290
*Sesn2*	Fwd: GCTGAAGACTGGCGAGCAC Rvs: CCAGAGAGTGGCAGTGGGTA	80	NM_144907.1
*Sesn3*	Fwd: GTGGTCCTCTTGGCTCACTATC Rvs: GCTGCTCACAGAGATTAGTCTGA	114	NM_030261.4
*Ddit3*	Fwd: CCTCGCTCTCCAGATTCCA Rvs: CTGTTTCCGTTTCCTAGTTCTTC	84	NM_007837
*Polr2b*	Fwd: GGTCAGAAGGGAACTTGTGGTAT Rvs: GCATCATTAAATGGAGTAGCGTC	197	NM_153798.2

### 2.5 Staining and Fluorescence Microscopy

C2C12 myotubes were washed twice with PBS and fixed with 3.7% formaldehyde (Sigma–Aldrich, Dorset, UK) for 10 min. The cells were then washed twice with PBS and permeabilised with 0.2% trition‐X100 (Sigma–Aldrich, Dorset, UK) in TBS for 30 min and then incubated for 1 h in the dark in TBS with DAPI (1:1000; Fisher Scientific) to visualise nuclei and rhodamine phalloidin (1:150; Invitrogen) to visualise the myotubes by staining the actin cytoskeletal structures. The cells were washed with 1 mL of TBS four times and imaged using a fluorescence microscope (Leica DM 2500, Leica Microsystems, Milton Keynes, UK) with 20× magnification. The width of myotubes was measured manually with Fiji Image J software (Schindelin et al. 2012). A myotube was defined as a single elongated structure containing three or more nuclei. To determine myotube width, three measurements were taken perpendicular to the long axis of the myotube, and the average value was taken as the width. A total of 20–30 images were captured and quantified per condition for each experiment, equating to a total of more than 200 myotubes per condition.

### 2.6 Statistical Analysis

Normality of distribution and homogeneity of variance were determined using Shapiro–Wilk tests and Levene's tests, respectively. The mRNA expressions of *Ddit3* and *Sesn2* across all time points, protein levels of sestrins with chemical treatments and protein levels of sestrin 2 and ATF4 with 4PBA treatment were analysed using one‐way ANOVA followed by Bonferroni adjustment for main effect comparison or Kruskal–Wallis tested followed by Mann–Whitney tests with Bonferroni correction where data were not normally distributed. The protein levels of ATF with chemical treatments and the basal characteristics of sestrin2 silencing in C2C12 myotubes were analysed with unpaired *t*‐test or Mann‐Whitney tests if data were not normally distributed. mTORC1 signalling in sestrin2 silenced cells were analysed using two‐way ANOVA with Bonferroni corrected pairwise comparison. All data are presented as mean ± SEM and analysed using IBM SPSS Statistics version 24.

## 3 Results

### 3.1 Sestrin Response to Cellular Stress in C2C12 Myotubes

**FIGURE 1:**
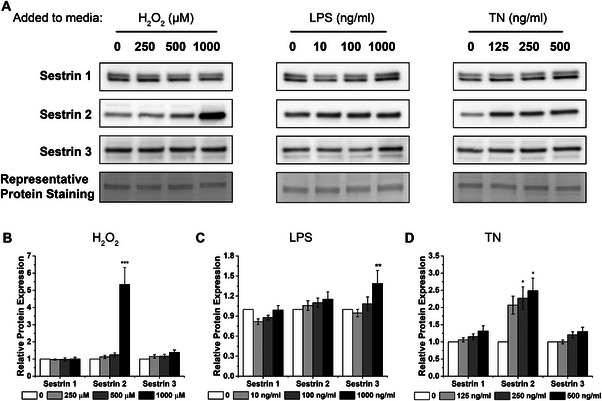
Induction of sestrin proteins in C2C12 myotubes following 24‐h incubation with hydrogen peroxide (H_2_O_2_), lipopolysaccharide (LPS) and tunicamycin (TN). (A) Representative immunobots of sestrin 1, 2 and 3 in well differentiated C2C12 myotubes after 24 h incubation with 0, 250, 500 and 1000 µM H_2_O_2_, 0, 10, 100 and 1000 ng/mL LPS, and 0, 125, 250 and 500 ng/mL TN. (B) Quantification of relative sestrin expression after 24‐h H_2_O_2_ treatment. (C) Quantification of relative sestrin expression after 24‐h LPS treatment and (D) quantification of relative sestrin expression after 24‐h TN treatment. Values are normalised to representative protein staining and made relative to 0 (CON). *** *p *< 0.0001. ** *p *< 0.001. * *p *< 0.05. Data are expressed as means ± SEM, *n* = 8 from three experimental repeats.

To determine how sestrin proteins are altered in response to stresses associated with muscle wasting conditions, C2C12 myotubes were treated with Lipopolysaccharide (LPS), Tunicamycin (TN) and hydrogen peroxide (H_2_O_2_) to stimulate inflammation, ER stress and oxidative stress respectively and were compared with vehicle treated control (CON) cells. 24‐h treatments with stress inducing chemicals were chosen initially to maximise the respective stress responses. Sestrins 1 and 3 were unaffected by H_2_O_2_ and TN treatments (Figure 1A, B and D). However, sestrin2 protein expression was increased 5‐fold (*p *< 0.0001) compared with CON after incubation with 1000 µM of H_2_O_2_ (Figure 1A,B). In addition, the effect of H_2_O_2_ incubation on sestrin2 level was not dose‐dependent, as no increase in sestrin2 expression was observed with incubation at 250 and 500 µM (Figure 1B). Sestrin2 protein level also increased by more than 2‐fold across 125, 250 and 500 ng/mL of TN treatments compared with CON (*p *= 0.053, *p *= 0.016 and *p *= 0.004, respectively, Figure 1A,D). Unlike the H_2_O_2_ and TN treatment, LPS treatment has no effect on sestrin1 and 2 protein levels (Figure 1A,C). Instead, sestrin3 protein level was upregulated by 40% following 24 h of treatment with 1000 ng/mL LPS (*p *< 0.001) compared with CON, but no change in sestrin3 was observed at lower concentrations (Figure 1C).

**FIGURE 2:**
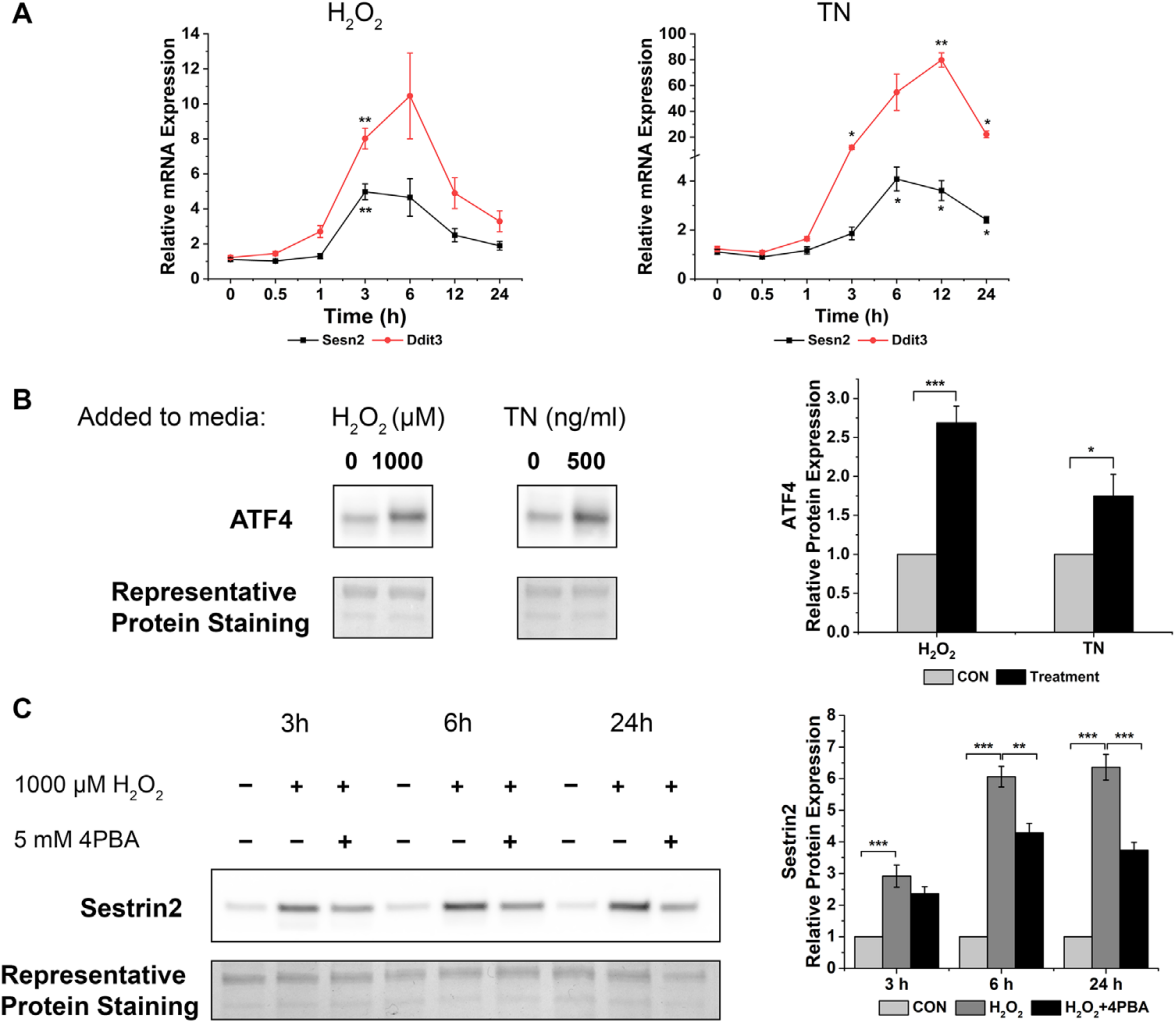
Increased sestrin 2 levels are associated with ER stress in C2C12 myotubes. (A) mRNA levels of sestrin2 and Ddit3 over a 24‐h time course following incubation with H_2_O_2_ or TN. (B) ATF4 protein levels after incubation with 1000 µM H_2_O_2_ and 500 ng/mL TN and (C) sestrin2 protein levels over a 24‐h time course following incubation with either with 1000 µM H_2_O_2_ alone or in combination with 5 mM 4‐sodium phenylbutyrate (4‐PBA). *** *p *< 0.0001. ** *p *< 0.001. * *p *< 0.05 compared to 0 h, or as indicated in figure. Data are expressed as means ± SEM, *n* = 6 from three experimental repeats.

High concentrations of H_2_O_2_ have been shown to enhance ER stress in myotube cultures (Pierre et al. 2014), and specific induction of sestrin2 has previously been observed in other cell types in response to ER stress and its ensuing cell death response (Ding et al. 2016), through the transcription factor ATF4. Indeed, ATF4 protein levels were induced significantly by both H_2_O_2_ (*p *< 0.001) and TN (*p *< 0.05, see Figure 2B), but not LPS (not shown), suggesting that this pathway may be implicated in the sestrin2 response to stress in myotubes.

Next, we measured *Sesn2* and *Ddit3* (a well‐described marker of excessive ER stress, which can lead to cell death) mRNA expression over a 24‐h time course following the addition of either 1000 µM H_2_O_2_ or 500 ng/mL TN, to understand if changes in ER stress coincide with sestrin2 induction. Compared to 0 h there was a 5‐fold increase in *Sesn2* expression after 3‐h incubation with H_2_O_2_ (*p *< 0.01), which remained elevated after 6‐h, although not significantly (*p *= 0.416), before returning to near basal levels after 24 h (see Figure 2A). The time course of *Ddit3* induction by H_2_O_2_ was similar to that of *Sesn2*, whereby *Ddit3* mRNA levels increased by 8‐fold (*p *< 0.01) and 10‐fold (*p *= 0.253) by 3 and 6 h, respectively, before the levels decreased back towards baseline (see Figure 2A). This indicates that H_2_O_2_ induces an ER stress response that coincides with *Sesn2* transcription. Similarly, in TN treated C2C12 myotubes the *Sesn2* expression started to increase after 3‐h incubation and peaked at 6 h with 4‐fold increase compared to 0 h (*p* < 0.05, Figure 2A). *Sesn2* mRNA levels remained statistically elevated at 12 and 24 h (*p *< 0.05). The expression of *Ddit3* increased significantly at 3, 12 and 24 h (*p *< 0.05) compared to 0 h following treatment with TN and although at 6 h the fold increase in *Ddit3* mRNA was nearly 50 times greater than 0 h, the variation in the data was too large to be statistically significant (*p *= 0.257). Collectively, the time‐dependent changes in *Ddit3* expression seemed to mirror *Sesn2* mRNA levels in response to TN and H_2_O_2_, strongly suggesting that ER stress and the downstream response is involved in sestrin 2 transcription within myotubes. DNA damage is also implicated in sestrin2 transcription, but mRNA expression of DNA damage response proteins *Brca1* and *Trp53* was unchanged following TN or H_2_O_2_ treatment (data not shown).

To further investigate the link between ER stress and sestrin 2 in C2C12 myotubes, 1000 µM H_2_O_2_ was co‐incubated with a chemical chaperone, sodium phenylbutyrate (4PBA), to assist in protein folding and dampen the ER stress response. Co‐incubation of H_2_O_2_ with 4PBA suppressed the increase in sestrin2 protein levels by 31% and 48% at 6 and 24‐h compared to treatment with H_2_O_2_ alone (*p *< 0.001, see Figure 2C). This further confirms that H_2_O_2_ induces ER stress in C2C12 myotubes which appears to contribute to the induction of sestrin2.

### 3.2 Silencing Sestrin 2 Has Little Effect on Myotube Anabolism Under Basal Conditions

**FIGURE 3:**
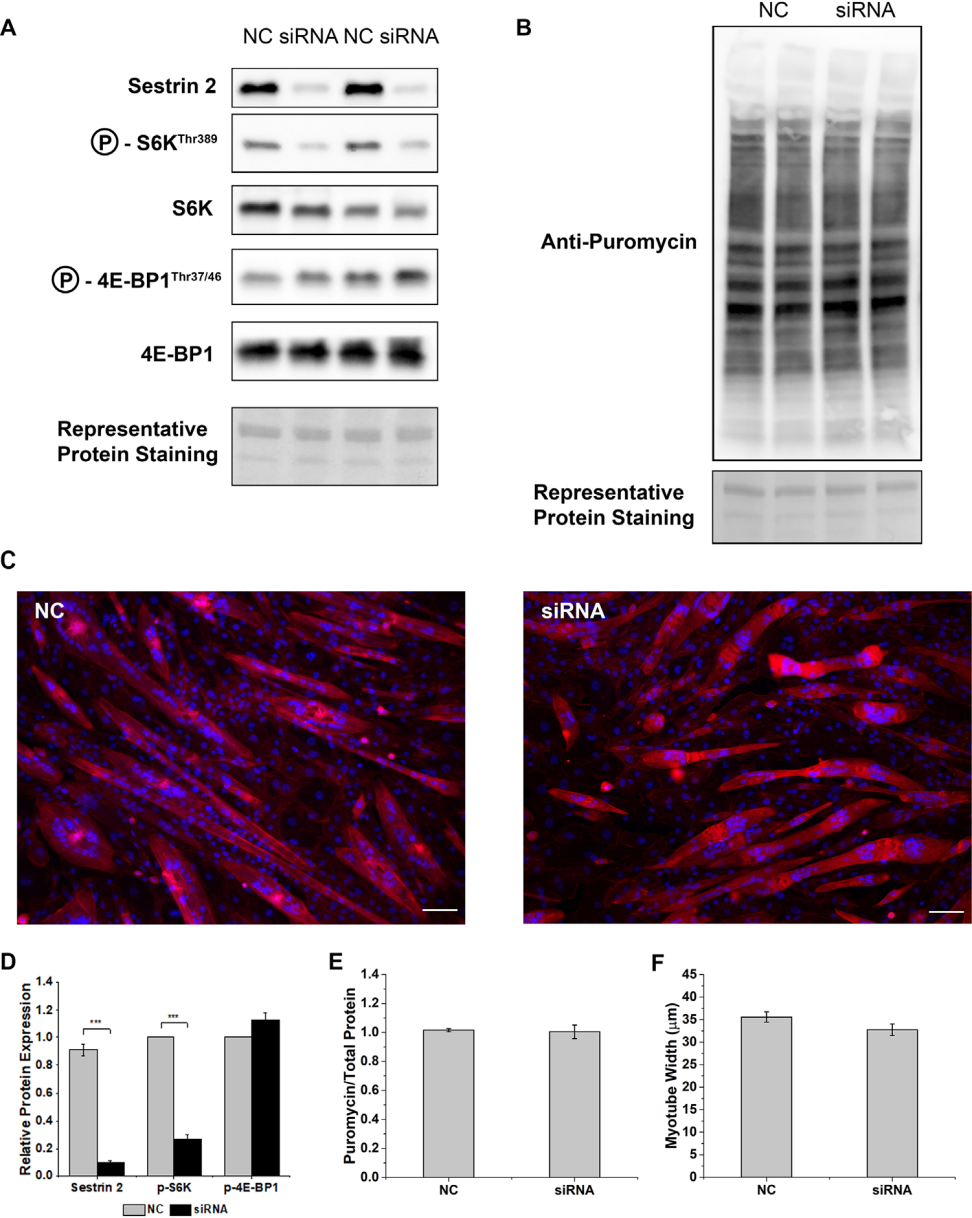
Silencing sestrin2 in C2C12 myotubes dampens S6K1 phosphorylation but has no impact on acute measures of protein synthesis or myotube size. (A) Representative immunoblots of sestrin 2 and the mTORC1 signalling markers S6K1 and 4E‐BP1 in control (NC) and sestrin 2 silenced (siRNA) C2C12 myotubes, (B) representative immunoblot indicating puromycin incorporation into nascent proteins in NC or siRNA C2C12 myotubes, (C) rhodamine phalloidin staining of the actin cytoskeleton and counterstain with Dapi for visualisation of cellular nuclei in NC and siRNA C2C12 myotubes, (D) quantification of relative protein levels of sestrin 2, phosphor‐S6K1 and phosphor 4E‐BP1 in NC and siRNA myotubes, (E) quantification of relative puromycin incorporation in NC and siRNA treated myotubes and (F) quantification of relative myotube width in NC and siRNA treated myotubes. *** *p *< 0.0001. Data are expressed as means ± SEM, *n* = 6 from three experimental repeats.

Sestrins are well reported to regulate mTORC1 signalling in several cell types, but little data exists regarding sestrin2 function in muscle cells. Therefore, we next silenced sestrin2 using siRNA and examined markers of mTORC1 activation, along with protein synthesis and myotube size which lie downstream of mTORC1. Sestrin2 protein expression was suppressed by 89% after 24 h transfection with siRNA compared to NC (*p *< 0.05; Figure 3A,D), with no off‐target effects on sestrin1 and 3 protein levels (Figure S3). Surprisingly, siRNA mediated silencing of sestrin2 was associated with reduced S6K1 phosphorylation by 43% in C2C12 myotubes (*p *< 0.0001), with no difference in phosphorylation of 4E‐BP1 (Figure 3A,D). There was no difference in total S6K1 and 4E‐BP1 between NC and siRNA groups. However, despite these reductions in S6K1 there was no effect of sestrin2 silencing on protein synthesis as indicated by puromycin incorporation into nascent proteins (Figure 3B,E). Furthermore, 24 h after siRNA mediated sestrin2 silencing there was no difference in myotube size between silenced and NC conditions (Figure 3C,F) suggesting that sestrin2 is not essential for acute maintenance of myotubes.

### 3.3 mTORC1 Response to Amino Acid Deprivation Is Unchanged in Sestrin 2 Silenced Myotubes

**FIGURE 4:**
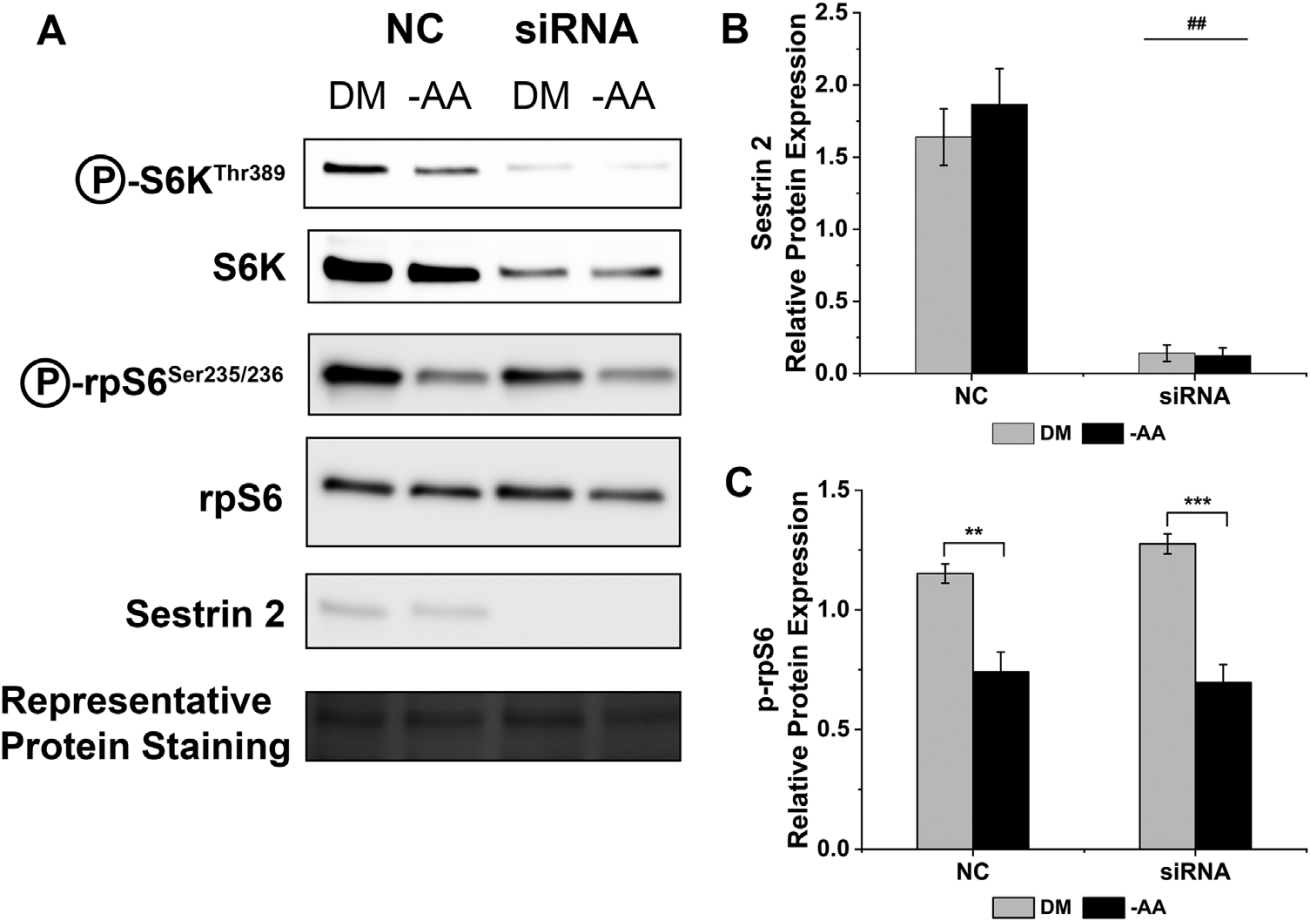
Amino acid and serum restriction reduces phosphorylation of ribosomal protein S6 in control and sestrin 2 siRNA myotubes. (A) Representative immunoblot indicating rpS6^ser235/236^ phosphorylation, rpS6 and setrin2 in NC and siRNA C2C12 myotubes in differentiation media (DM) or amino acid and serum restricted (–AA) media, (B) quantification of sestrin 2 protein levels in NC and siRNA C2C12 myotubes in DM and –AA media and (C) quantification rpS6^ser235/236^ phosphorylation in NC and siRNA C2C12 myotubes in DM and –AA media. *** *p *< 0.0001. ** *p *< 0.001. Data are expressed as means ± SEM, *n* = 8 for rpS6 and *n* = 5 for sestrin2, from three experimental repeats.

To assess the importance of sestrin2 for mTORC1 sensitivity to AAs, we next conducted amino acid/serum deprivation experiments in sestrin2 silenced C2C12 myotubes. Since S6K1 phosphorylation was blunted by silencing sestrin2, in this experiment we also chose to measure phosphorylation of rpS6 as a measure of mTORC1 activity as it is a well‐known surrogate for mTORC1 signalling. Sestrin2 protein levels were unchanged in response to amino acid withdrawal (*p =* 0.530, Figure 4A,B), suggesting that sestrin2 content is not influenced by nutrient availability. rpS6 phosphorylation was decreased by 59% by amino acids/serum deprivation (*p* < 0.001), but interestingly this effect was not different between NC and sestrin2 silenced myotubes (*p* = 0.195, Figure 4A,C). There was no effect of siRNA or amino acid availability on total rpS6 levels.

## 4 Discussion

Muscle wasting such as occurs in disease, disuse or ageing is often associated with cellular stress (Sartori et al. 2021). Sestrins are a family of stress inducible proteins, and potentially important regulators of skeletal muscle size and health though their functions as cellular amino acid sensors and negative regulators of the mTORC1 signalling pathway (Kim et al. 2020; Parmigiani et al. 2014; Segalés et al. 2020; Wolfson et al. 2016). However, currently it is not well known how sestrin proteins respond to cellular stresses linked to muscle loss. Furthermore, whilst sestrin1 is the most abundant sestrin protein in skeletal muscle (Peeters et al. 2003), the role of sestrin2 in regulation of muscle size and mTORC1 sensing of amino acids is less clear. In the present study, we found that sestrin2 was uniquely stress responsive and appears to coincide with endoplasmic reticulum (ER) stress in C2C12 myotube cultures. Surprisingly, subsequent experiments showed that siRNA silencing of sestin2 in myotubes reduced S6K1 phosphorylation (a downstream effector of mTORC1) but had no acute effect on protein synthesis or myotube size. There was also no effect of sestrin2 silencing on mTORC1 response to amino acid deprivation.

Although it has been established that sestrins 1 and 2 can protect muscle from atrophy under several conditions (Segalés et al. 2020; Yang, Xue, et al. 2021), the link between specific stresses associated with muscle wasting and sestrin levels are not clear. We found that sestrin2 was specifically stress sensitive and was induced by incubation of myotubes with hydrogen peroxide (H_2_O_2_) and Tunicamycin (TN), but not LPS. H_2_O_2_ has previously been shown to cause ER stress in C2C12 myotubes and activate the PERK arm of the unfolded protein response (UPR) (Pierre et al. 2014). In turn, the PERK arm of the UPR (but not IRE‐1 or ATF‐6) is required for sestrin2 induction following rotenone treatment in muscle cells (Yang, Xue, et al. 2021), which when coupled with the fact that TN enhanced sestrin2, led us to speculate that ER stress may principally be involved in in upregulation of sestrin2 in our experiments. Indeed, when mapped over 24‐h, the profile of *Sesn2* mRNA coincided with that of the ER stress marker *Ddit3* (CHOP protein) in response to both H_2_O_2_ and TN and could be inhibited by approximately half by the ER‐stress inhibitor 4‐PBA, confirming the contribution of ER stress and the UPR to sestrin2 induction in muscle cells.

Whilst our data confirms that sestrin2 (but not other sestrin family members) is sensitive to ER stress, we are not the first to show this unique sestrin2 response, as it has previously been reported in other cell types that chemical inducers of ER stress, or nutrient/energetic stimulation of ER stress results in specific increases in sestrin2 via the PERK arm of the UPR (Ding et al. 2016; Park et al. 2014). This unique induction of sestrin2 likely relates to its important role in preventing cell death. Indeed, sestrin2 is protective against several types of cell death that result from chronic or excessive ER stress (Ben‐Sahra et al. 2013; Ding et al. 2016; Saveljeva et al. 2016). Our experiments showed that H_2_O_2_ and TN caused a large upregulation of *Ddit3* mRNA levels, which is a protein related to ER stress induced apoptosis (Hetz 2012), and this coincided with sestrin2 induction over 24‐h. As such, it is likely that chronic ER stress derived from TN or high H_2_O_2_ concentrations stimulated sestrin2 as a cytoprotective response. Indeed, muscle fibre death is a key contributing factor to muscle wasting conditions associated with cellular stress such as immobilisation, denervation and ageing (Siu 2009). Recently, Yang, Xue, et al. (2021) and Yang, Guo, et al. (2021) showed that muscle denervation resulted in ER stress dependent elevations in sestrin2 levels (but not sestrin 1 or 3) and knockout of sestrin2 during denervation exacerbated apoptosis and muscle loss. Collectively, it can therefore be surmised that during muscle wasting, ER stress is a key stimulus for sestrin2, which likely plays an important role in preventing muscle loss.

In contrast to sestrin2, we observed no change in sestrin1 across treatments and a small but significant elevation in sestrin3 in myotubes treated with 1000 ng/mL LPS, which stimulates an inflammatory response. Sestrins 1 and 2 share several functions (Chen et al. 2022), but whereas sestrin2 is known to respond to several stresses, genotoxic stress appears to be required for sestrin1 induction (Velasco‐Miguel et al. 1999). Since we saw no increase in *Brca1* or *Trp53* gene expression following H_2_O_2_ or TN treatments, it is likely that the stresses in the present study were not sufficient to induce significant DNA damage. Whilst less is known of the function of sestrin3, it appears to play a role in protecting against insulin resistance (Lee et al. 2013; Tao et al. 2015) and is elevated in type II diabetics (Nascimento et al. 2013). Since diabetes and insulin resistance are associated with chronic inflammation (Ferroni et al. 2004) and muscle wasting (Shen et al. 2022), future research should investigate the role of sestrin3 in protection against muscle wasting related to inflammation.

One of the best‐established functions of sestrin proteins is their regulation of mTORC1 signalling (Chantranupong et al. 2014; Parmigiani et al. 2014; Peng et al. 2014). In turn, since mTORC1 controls protein synthesis and muscle growth (Bodine 2022) through its downstream effectors S6K1 and 4E‐BP1, the relationship between sestrins and mTORC1 is important to understand in the context of muscle wasting. However, in skeletal muscle, sestrin2 is lowly expressed, and its impact on mTORC1 and muscle size is not well studied. Indeed, based on previous research depicting sestrin2 as a negative regulator of mTORC1 in other cell types (Budanov and Karin 2008; Parmigiani et al. 2014; Peng et al. 2014), we expected silencing of sestrin2 to enhance mTORC1 signalling and cell size. However, in our hands silencing of sestrin2 resulted in dampened S6K1 phosphorylation by approximately 80%, and whilst we cannot account for this large suppression of S6K1, others have also observed a decrease in S6K1 phosphorylation of approximately 50% with siRNA mediated sestrin2 silencing in HepG2 cells (Yang, Guo, et al. 2021), which was similar to our data, and therefore not entirely unprecedented. Despite the attenuation of S6K1, we found no impairment in basal protein synthesis or myotube size, suggesting that knockdown of sestrin2 did not acutely influence muscle anabolism under basal conditions (i.e., unstressed and fed cells). It is possible that phosphorylation of 4E‐BP1 exerts preferential influence over protein synthesis (Laplante and Sabatini 2009), and since no change in 4E‐BP1 phosphorylation was observed in the present experiments, this may account for the lack of alteration in protein synthesis or myotube size. Longer post‐transfection incubation periods might be required to observe any chronic changes in basal protein synthesis or myotube size and maintenance, as previous studies have observed changes in cell size 48‐h after sestrin gene transfer (Budanov and Karin 2008; Parmigiani et al. 2014). Nonetheless, these data broadly align with previous work in adult mice, indicating a limited role for sestrin2 in regulating muscle size or function under basal conditions (Segalés et al. 2020), but do not preclude the ability of sestrin2 to regulate mTORC1 under conditions of muscle loss to help preserve muscle mass (Segalés et al. 2020).

The principal way in which sestrins regulate mTORC1 signalling is through acting as sensors for the amino acid leucine, whereby upon binding leucine, sestrin2's inhibition over GATOR2 is relinquished, enabling mTORC1 activation (Chantranupong et al. 2014; Wolfson et al. 2016). As such, previous work in HEK293 cells has illustrated that loss of sestrin2 desensitises mTORC1 to amino acid withdrawal (Chantranupong et al. 2014). However, we found that removal of amino acids from the culture media attenuated mTORC1 signalling similarly in both control and sestrin2‐silenced myotubes. Since silencing sestrin2 unexpectedly inhibited S6K1 under basal conditions, we used rpS6 as a primary readout of mTORC1 for this experiment, which, in a similar fashion to 4E‐BP1, was unaffected by sestrin2 silencing. It is possible that, unlike other cell types, sestrin2 is not an important contributor to amino acid sensing in skeletal muscle. Indeed, this possibility is in keeping with work by Xu et al. (2019) who found that in rodent muscle leucine had no effect on the sestrin 2‐GATOR2 interaction but instead causes dissociation of sestrin1 from GATOR2. In turn, this suggests that sestrin1 (and not sestrin2) is the predominant amino acid sensor regulating mTORC1 in muscle, which makes sense since sestrin1 is highly expressed in this tissue type (Peeters et al. 2003; Xu et al. 2019). However, it should be noted that overexpression of sestrin1 did not significantly alter mTORC1 sensitivity to amino acid feeding in rodent muscle (Xu et al. 2019), and as such, the mechanisms linking amino acid availability to mTORC1 signalling in skeletal muscle require further investigation.

In summary, the present experiments reveal that sestrin2 is uniquely responsive to stress associated with muscle wasting, and in particular ER stress appears to be important in inducing sestrin2 expression. However, silencing sestrin2 had no acute effect on myotube size, protein synthesis or mTORC1 sensitivity to amino acid withdrawal. Coupled with the existing literature, this data suggests that sestrin2 has an important role in protecting skeletal muscle in response to stress but plays less of a role in mTORC1 regulation and nutrient sensing in muscle cells in an unstressed/basal state.

## Funding

This work was supported in part by a Rank Prize Funds New Investigator Award to NRWM.

## Conflicts of Interest

The authors declare no conflicts of interest.

## Supporting information




**Supporting File 1**: boc70040‐sup‐0001‐FigureS1.png

**Supporting File 2**: boc70040‐sup‐0002‐FigureS2.png

**Supporting File 3**: boc70040‐sup‐0003‐FigureS3.png

## Data Availability

The data that support the findings of this study are available from the corresponding author upon reasonable request.
