# Supplementary figures and images for: Sestrin2 is Induced Upon Cellular Stress but Has No Effect on Myotube Size or Amino Acid Sensing in C2C12 Myotubes
Source: Biol Cell. 2025 Nov 6;117(11):e70040. doi: 10.1111/boc.70040 (PMC12590934; doi:10.1111/boc.70040)

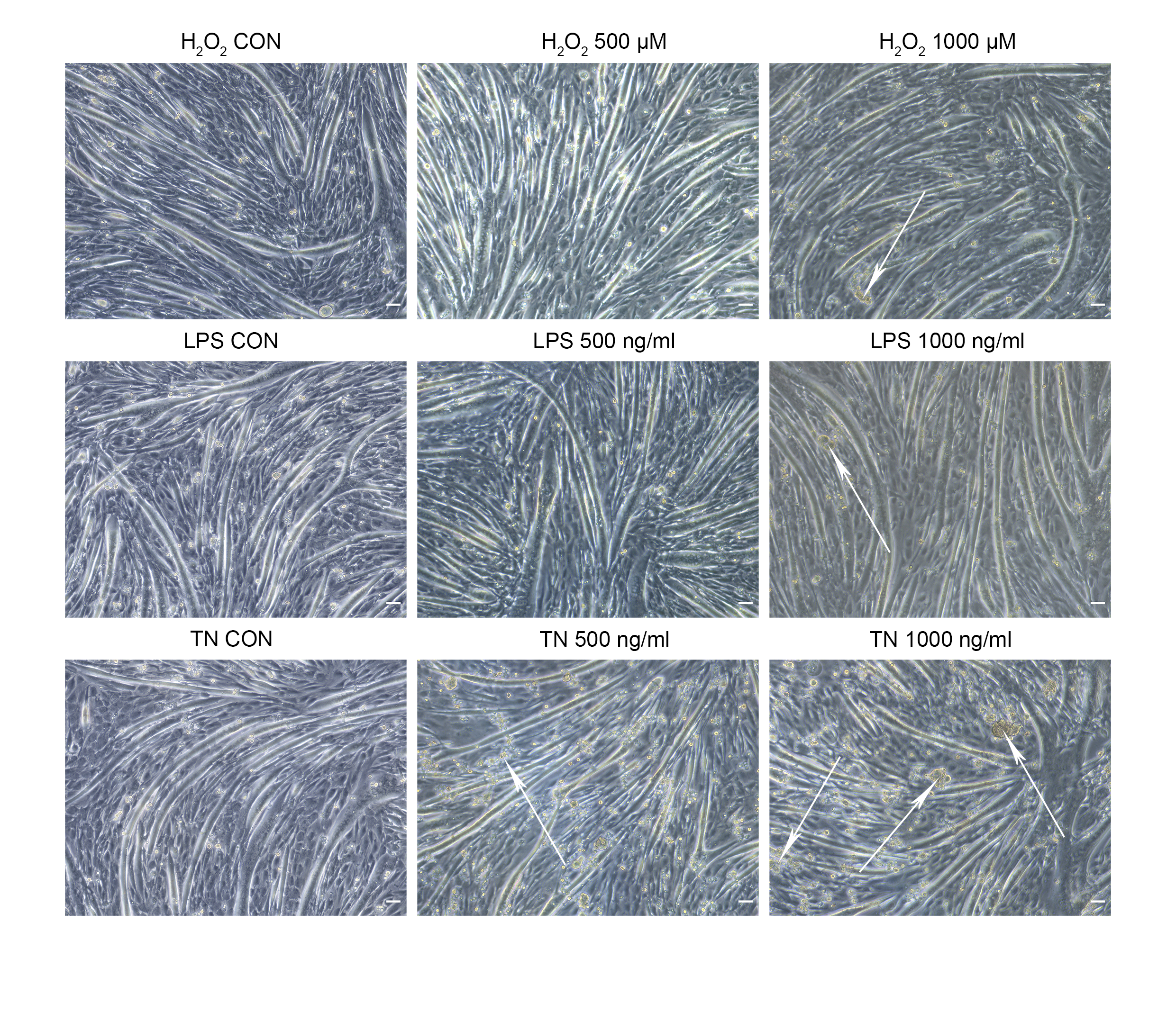

Supplement: Supplementary file 1 — Supporting File 1: boc70040‐sup‐0001‐FigureS1.png [file BOC-117-e70040-s001.png]

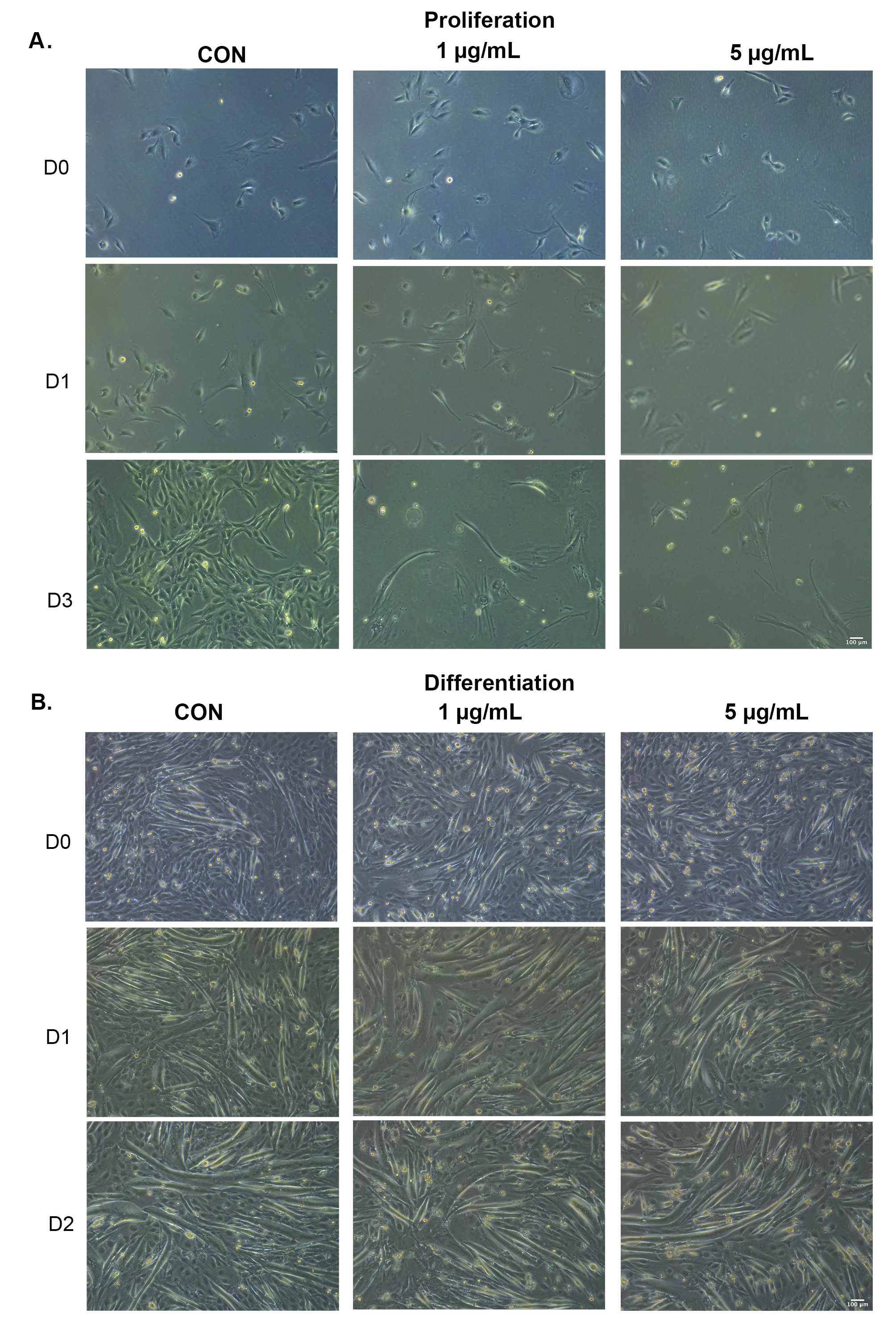

Supplement: Supplementary file 2 — Supporting File 2: boc70040‐sup‐0002‐FigureS2.png [file BOC-117-e70040-s003.png]

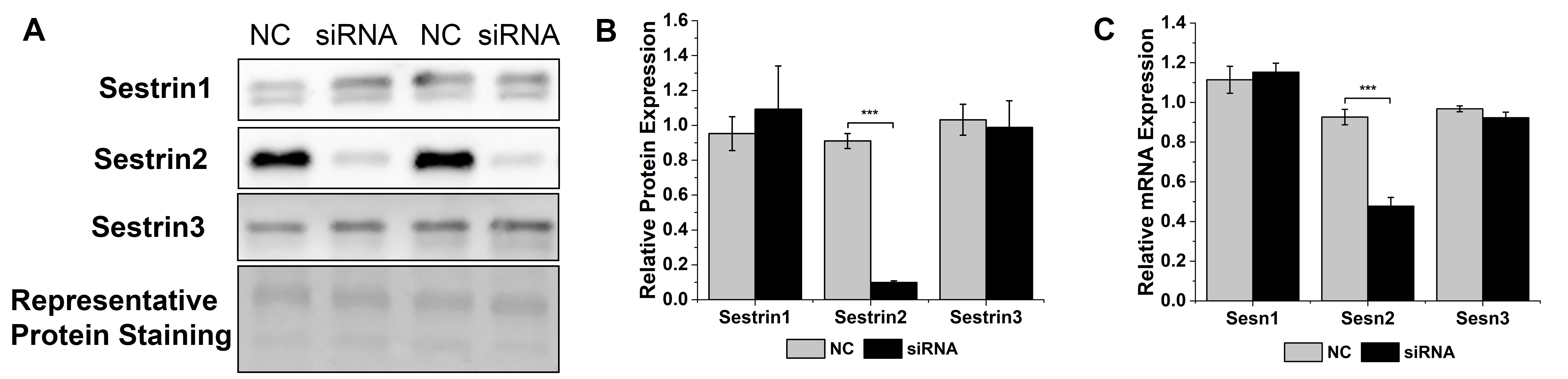

Supplement: Supplementary file 3 — Supporting File 3: boc70040‐sup‐0003‐FigureS3.png [file BOC-117-e70040-s002.png]
